# Supplementary material for: Molecular crowding effects on protein stability in a bacterial proteome
Source: Sci Rep. 2026 Jan 21;16:5908. doi: 10.1038/s41598-026-35990-9 (PMC12894976; doi:10.1038/s41598-026-35990-9)
Supplement: Supplementary file 2 — Supplementary Material 2 [file 41598_2026_35990_MOESM2_ESM.zip › AllTheTPPData/P1vP8/Panobinostat_Vignette/Melting_Curves/meltCurve_Q0KAE4.pdf]

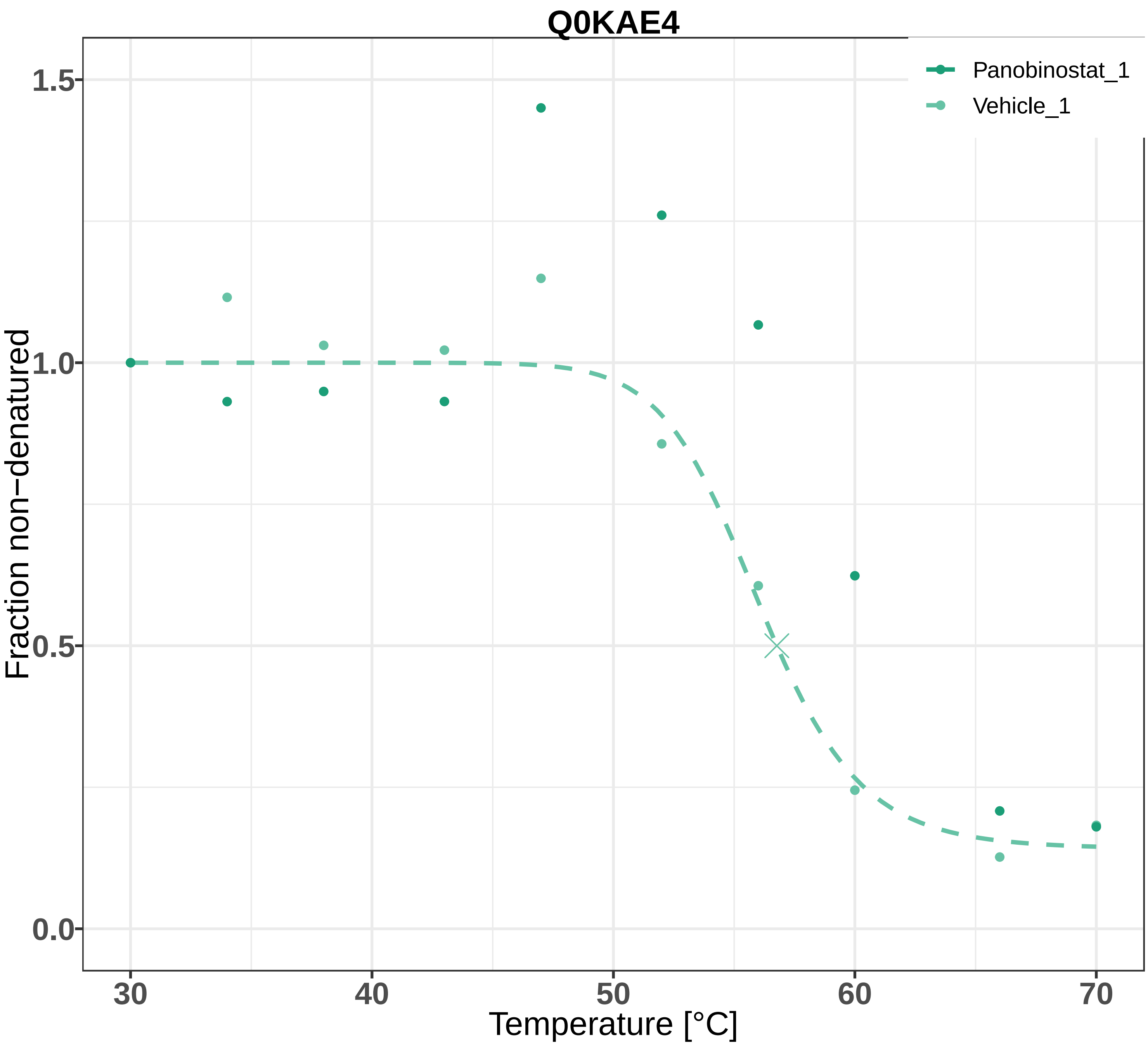

	meltPoint	slope	plateau	R2
Panobinostat_1	–	–	–	–
Vehicle_1	56.77	−0.1	0.14	0.97
